# Supplementary material for: Predicting Concentrations of Mixed Sugar Solutions with a Combination of Resonant Plasmon-Enhanced SEIRA and Principal Component Analysis
Source: Sensors (Basel). 2022 Jul 26;22(15):5567. doi: 10.3390/s22155567 (PMC9329749; doi:10.3390/s22155567)
Supplement: Supplementary file 1 [file sensors-22-05567-s001.zip › sensors-1778242-supplementary.pdf]

PREDICTING CONCENTRATIONS OF MIXED SUGAR SOLUTIONS WITH A COMBINATION OF RESONANT PLASMON-ENHANCED SEIRA AND PRINCIPAL COMPONENT ANALYSIS

Diana Pfezer, Julian Karst, Mario Hentschel, and Harald Giessen

4th Physics Institute and Research Center SCoPE, University of Stuttgart, Pfaffenwaldring 57, 70569 Stuttgart, Germany

Supplementary Information

1. Reproducibility
   1.1. Mixtures with same concentration have not the same SC values (see Fig. S1).
2. Mixtures with low concentrations
   2.1. Glucose and fructose mixtures down to 5:5 g/l (see Fig. S2).
3. Adaptive method for quantitative estimation of glucose and galactose, glucose and lactose, glucose and maltose concentrations in aqueous solutions based on infrared nanoantenna optics
   3.1. This method works well for molecules which have vibrational modes appearing not at the same wavenumbers, such as glucose and galactose (see Fig. S3).
   3.2. When the vibrational modes are getting closer, then the results are getting worse, especially, for glucose and maltose. (see Fig. S4 and S5).

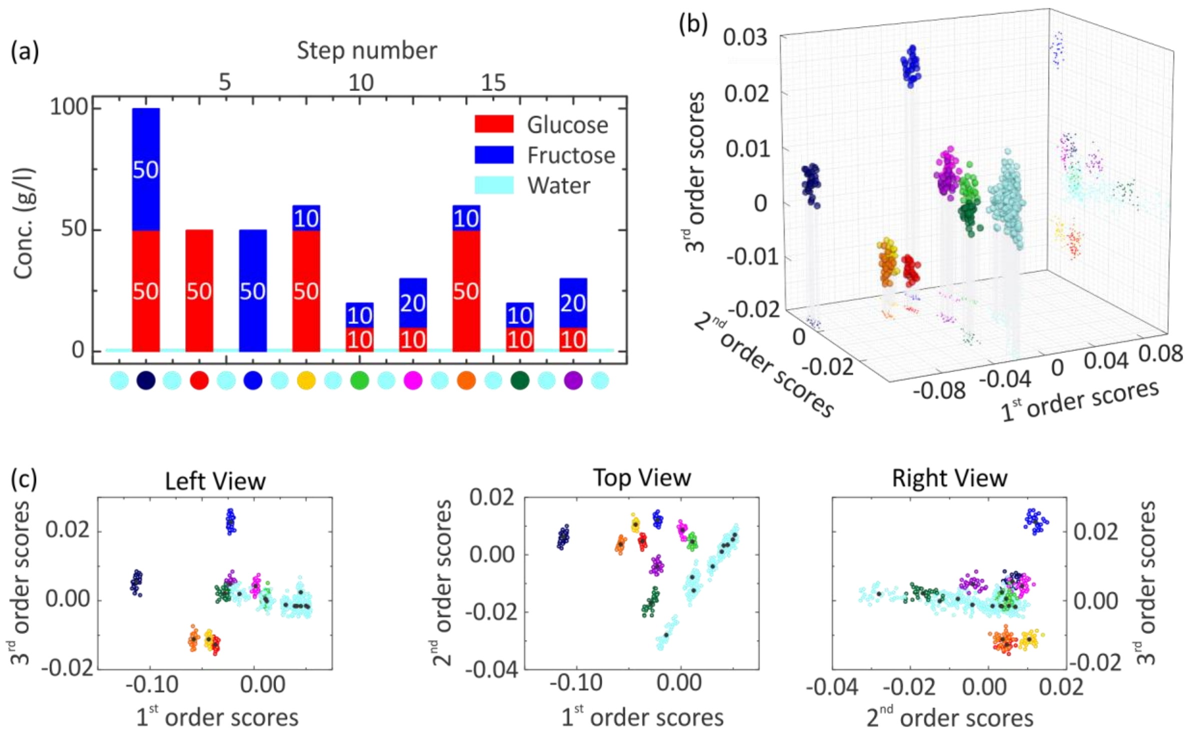

Figure S1 Measurement of mixtures to investigate reproducibility. (a) Measurement cycle of pure and mixed aqueous sugar solutions of glucose and fructose as well as water. The red bars indicate the concentration of glucose in the solution and the blue bars of fructose. Between an aqueous sugar solution pure water solutions are measured. 30 spectra are measured for each step. (b,c) PCA of the measured spectra from (a). SCs of the 1st, 2nd, and 3rd order are plotted against each other in a 3D plot (b) and the corresponding 2D plots are generated (c). Each measurement step is color-coded, whereas water has the same color and the other solutions an individual color depicted at the bottom of the measurement cycle in (a). As a result, cluster are obtained for each measured solution. The identification and the differentiation of the different probed aqueous solutions is possible with a 3D plot of the 1st, 2nd, and 3rd order SCs. The measurement with mixtures are not reproducible because same solutions do not exhibit the same weight of the SCs.

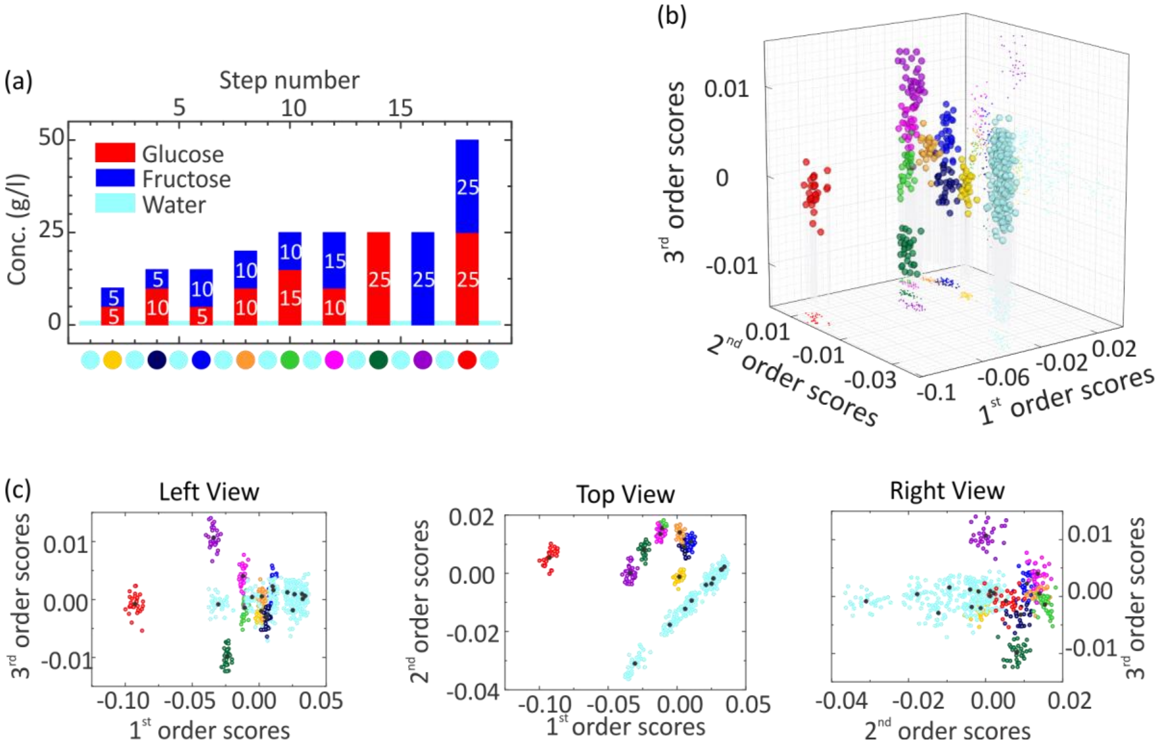

Figure S2. Measurement of mixtures with low concentrations. (a) Measurement cycle of pure and mixed aqueous sugar solutions of glucose and fructose as well as water (30 spectra are taken for each step). The red bars indicate the concentration of glucose, the blue bars of fructose, the cyan bars indicate a pure water solution. (b,c) PCA of the measured spectra from (a). SCs of the 1st, 2nd, and 3rd order are plotted against each other in a 3D plot (b) and the corresponding 2D plots are generated (c). For each measurement step a cluster is obtained, which has an individual color depicted at the bottom of the measurement cycle in (a). All water measurements have the same color. The 3D plot of the 1st, 2nd, and 3rd order SCs allows the identification and differentiation of the various probed aqueous solutions. Mixtures with low concentrations down to 10 g/l can be detected.

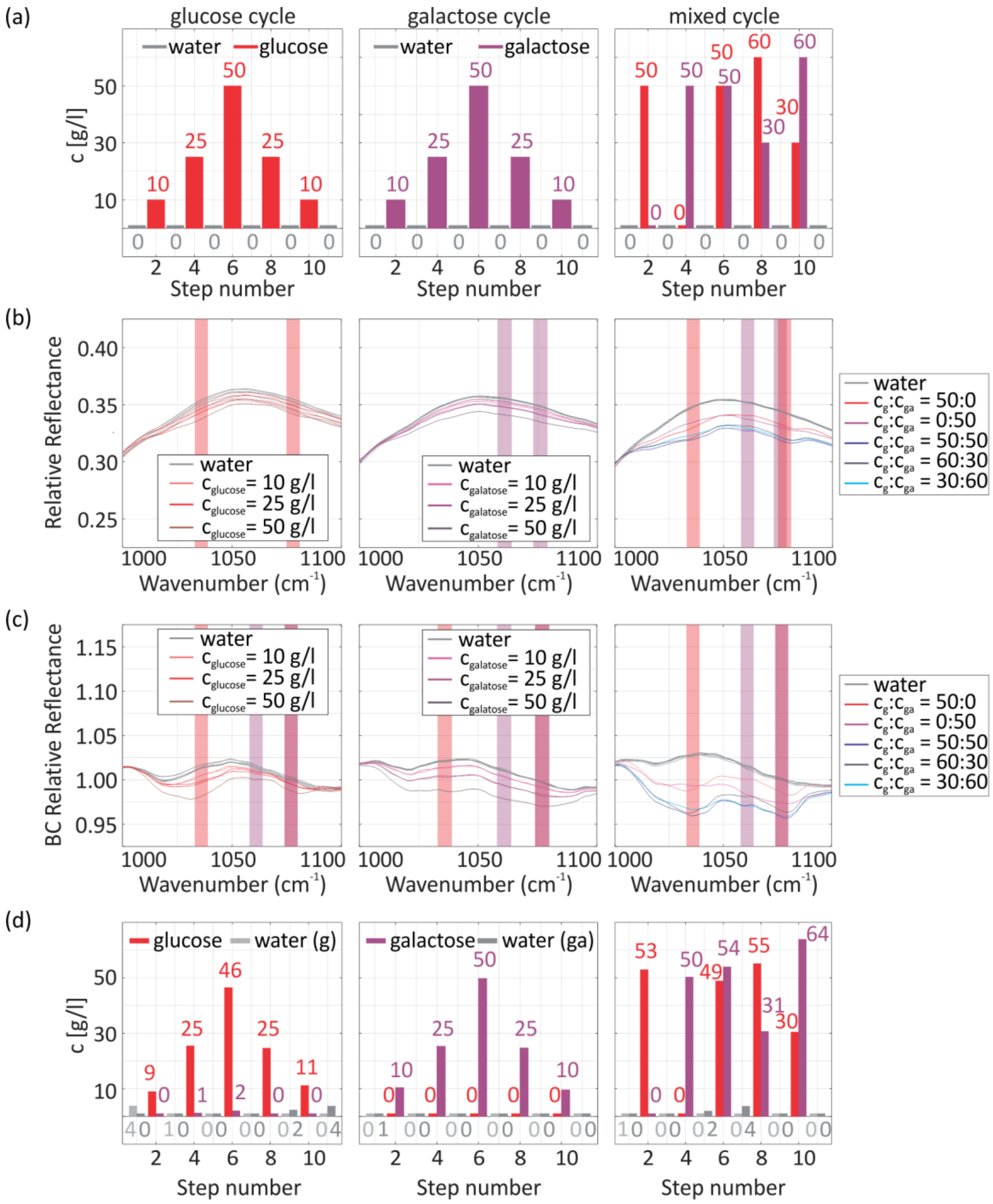

Figure S3. Quantitative estimation of glucose and galactose concentrations. (a) Measurement cycles of pure and mixed aqueous sugar solutions of glucose and galactose as well as water, whereas 30 spectra are taken for each step. The red bars indicate the concentration of glucose, the purple bars of galactose, the gray bars indicate a pure water solution. (b) For each step, the average of the 30 spectra are calculated and the resulting SEIRA reflectance spectra are depicted. The molecular vibrations of glucose and galactose are highlighted by the red bars and the purple bars, respectively. (c) Baseline-corrected vibrational spectra of the aqueous solutions are acquired with λ = 1250 and p = 0.5. A slightly modified version of the asymmetric least squares smoothing (ALSS) algorithm was

used.[1] (d) Adaptive method for quantitative estimation of glucose and galactose concentrations in aqueous solutions, whereby cycle-specific konstants *k*i as well as cubic polynomials φ_i and ψ_i are taken. The positions of the characteristic resonance frequencies are at 1036 cm^{-1} for glucose, 1061 cm^{-1} for galactose and 1077 cm^{-1} for glucose and galactose. The adaptive method from Schuler et al. was used and all MATLAB related files are taken from there.[2] The quantification of the glucose and galactose concentrations works well for the pure and mixed aqueous solutions.

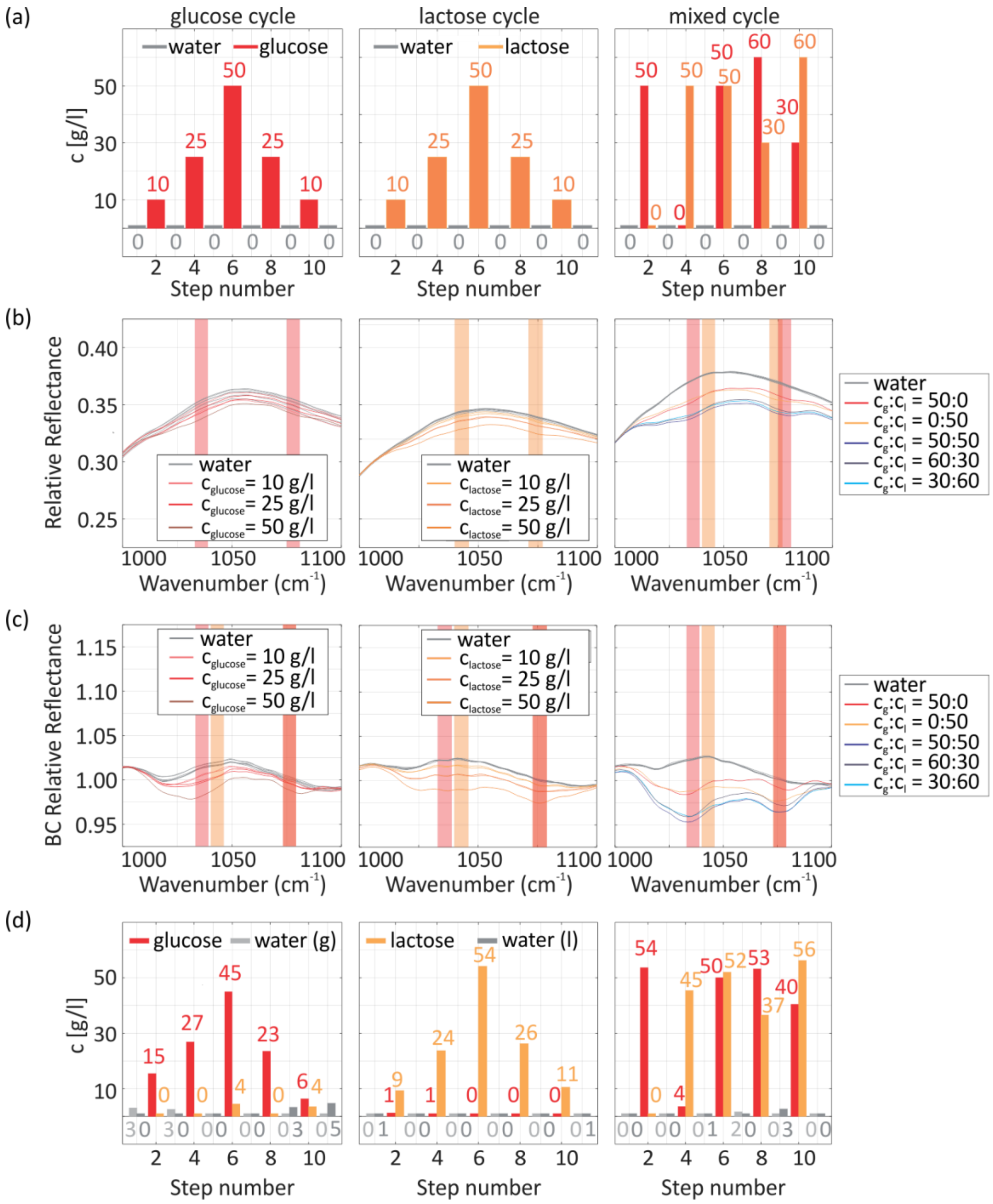

Figure S4. Quantitative estimation of glucose and lactose concentrations. (a) Measurement cycles of pure and mixed aqueous sugar solutions of glucose and lactose as well as water, whereas 30 spectra are taken for each step. The red bars indicate the concentration of glucose, the orange bars of galactose, the gray bars indicate a pure water solution. (b) For each step, the average of the 30 spectra are calculated and the resulting SEIRA reflectance spectra are depicted. The molecular vibrations of glucose and lactose are highlighted by the red bars and the orange bars, respectively. (c) Baseline-corrected vibrational spectra of the aqueous solutions are acquired with λ = 500 and p = 0.25. A slightly modified version of the ALSS algorithm was used.[1] (d) Adaptive method for

quantitative estimation of glucose and lactose concentrations in aqueous solutions, whereby cycle-specific konstants *k*i as well as cubic polynomials φ_i and ψ_i are taken. The positions of the characteristic resonance frequencies are at 1036 cm^{-1} for glucose, 1043 cm^{-1} for lactose and 1076 cm^{-1} for glucose and galactose. The adaptive method from Schuler et al. was used and all MATLAB related files are taken from there.[2] The quantification of the glucose and lactose concentrations still works well for the pure and mixed aqueous solutions. The results are slightly worse than for the glucose and galactose case. One possible reason is that the vibrational modes are closer compared to the glucose and galactose case.

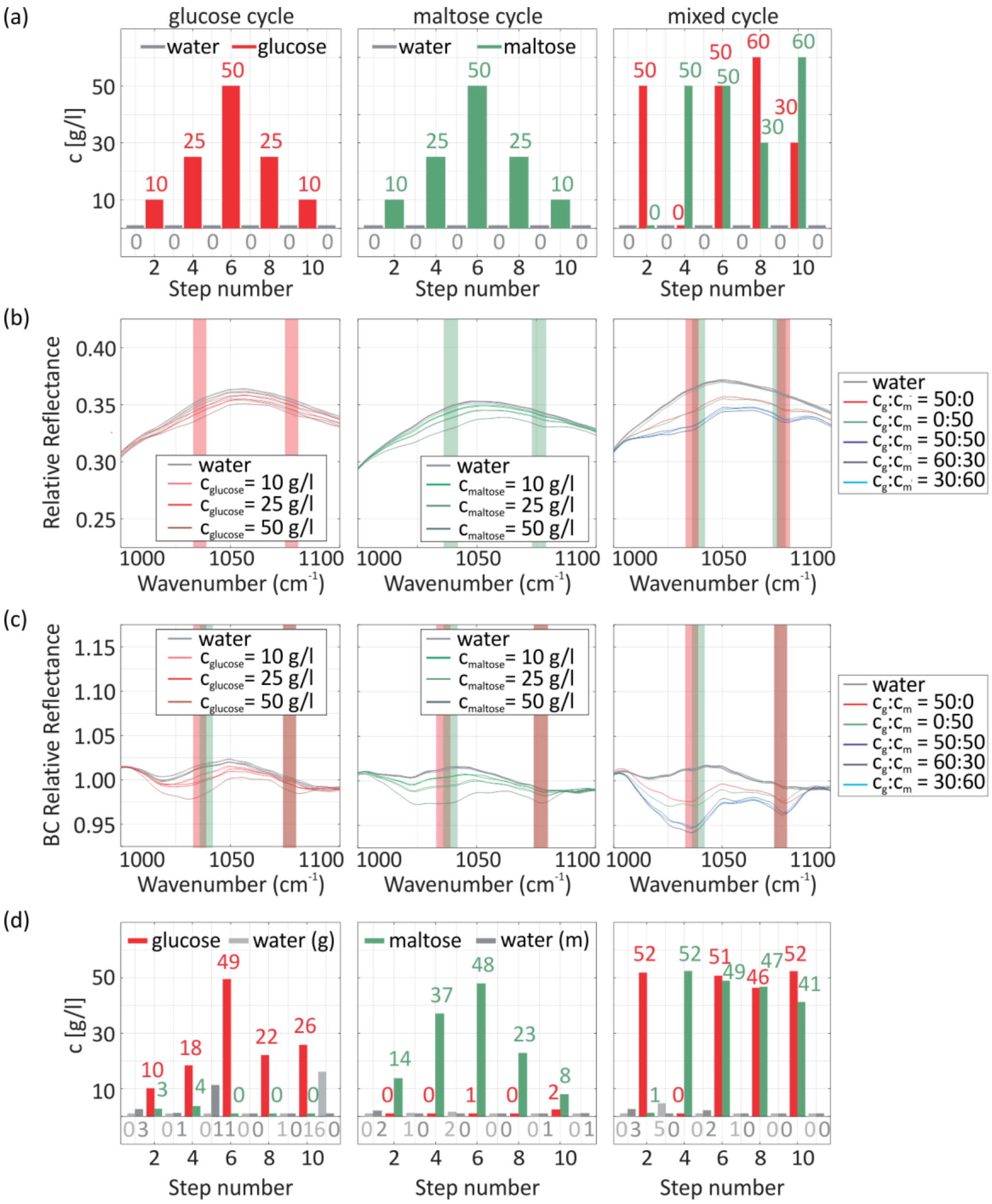

Figure S5. Quantitative estimation of glucose and maltose concentrations. (a) Measurement cycles of pure and mixed aqueous sugar solutions of glucose and maltose as well as water, whereas 30 spectra are taken for each step. The red bars indicate the concentration of glucose, the green bars of maltose, the gray bars indicate a pure water solution. (b) For each step, the average of the 30 spectra are calculated and the resulting SEIRA reflectance spectra are depicted. The molecular vibrations of glucose and lactose are highlighted by the red bars and the green bars, respectively. (c) Baseline-corrected vibrational spectra of the aqueous solutions are acquired with λ = 500 and p = 0.99. A slightly modified version of the ALSS algorithm was used.[1] (d) Adaptive method for

quantitative estimation of glucose and maltose concentrations in aqueous solutions, whereby cycle-specific konstants *k*i, linear polynomials φ_i and cubic polynomials ψ_i are taken. The positions of the characteristic resonance frequencies are at 1036 cm^{-1} for glucose, 1039 cm^{-1} for maltose and 1077 cm^{-1} for glucose and maltose. The adaptive method from Schuler et al. was used and all MATLAB related files are taken from there.[2] The quantification of the glucose and maltose concentrations works not good for the pure and mixed aqueous solutions. The results are much worse than the two previous cases. One possible reason is that the vibrational modes are near the same wavenumber.

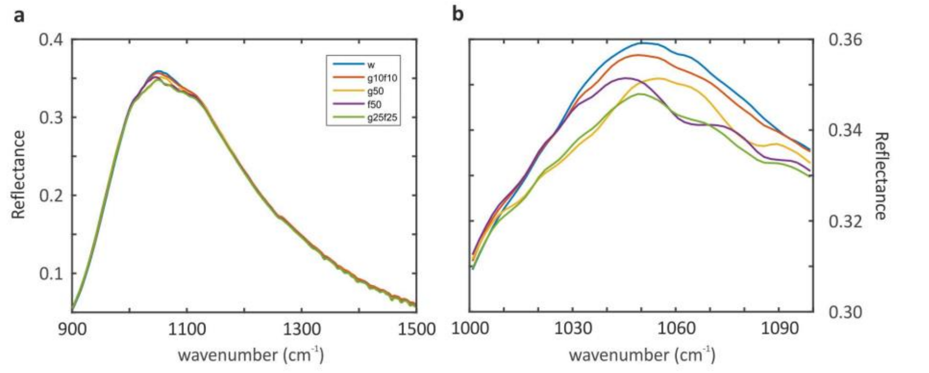

Figure S6. Selected raw reflection spectra of the measurements shown in Figure 4 of the main text. (a) Plasmonic resonance with imprinted vibrational features for pure water, 10 g/l glucose and fructose, 50 g/l glucose, 50 g/l fructose and a mixture of 25 g/l fructose and glucose. (b) Close-up of the region of interest for the vibrational modes.

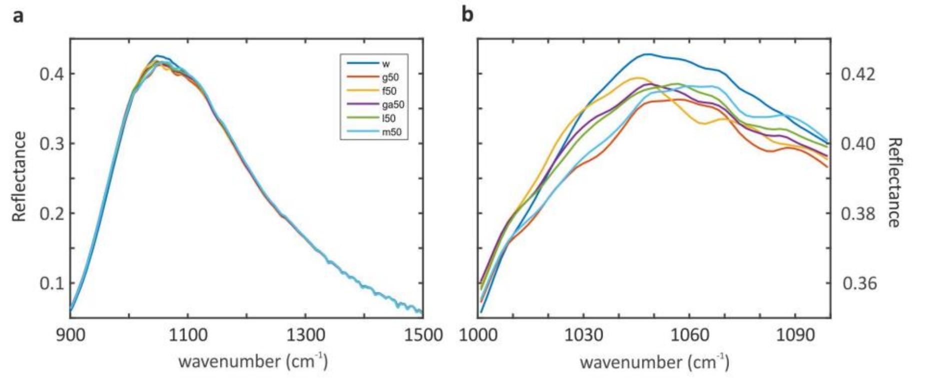

Figure S7. Selected raw reflection spectra of the measurements shown in Figure 5 of the main text. (a) Plasmonic resonance with imprinted vibrational features for pure water, and solution of 50 g/l glucose, fructose, galactose, lactose, and maltose each. (b) Close-up of the region of interest for the vibrational modes.

References

(1) Eilers, P. H. C.; Boelens, H. F. M. Baseline Correction with Asymmetric Least Squares Smoothing. *Leiden Univ. Med. Cent. Rep.* **2005**, *1*, 5.

(2) Schuler, B.; Kühner, L.; Hentschel, M.; Giessen, H.; Tarín, C. Adaptive Method for Quantitative Estimation of Glucose and Fructose Concentrations in Aqueous Solutions Based on Infrared Nanoantenna Optics. *Sensors (Switzerland)* **2019**, *19* (14). https://doi.org/10.3390/s19143053.
